# Scalable prediction of compound-protein interactions using minwise hashing

**DOI:** 10.1186/1752-0509-7-S6-S3

**Published:** 2013-12-13

**Authors:** Yasuo Tabei, Yoshihiro Yamanishi

**Affiliations:** 1 PRESTO, Japan Science and Technology Agency, Kawaguchi, Saitama 332-0012, Japan; 2 Division of System Cohort, Medical Institute of Bioregulation, Kyushu University, 3-1-1 Maidashi, Higashi-ku, Fukuoka, Fukuoka 812-8582, Japan; 3 Institute for Advanced Study, Kyushu University, 6-10-1, Hakozaki, Higashi-ku, Fukuoka, Fukuoka 812-8581, Japan

## Abstract

The identification of compound-protein interactions plays key roles in the drug development toward discovery of new drug leads and new therapeutic protein targets. There is therefore a strong incentive to develop new efficient methods for predicting compound-protein interactions on a genome-wide scale. In this paper we develop a novel chemogenomic method to make a scalable prediction of compound-protein interactions from heterogeneous biological data using minwise hashing. The proposed method mainly consists of two steps: 1) construction of new compact fingerprints for compound-protein pairs by an improved minwise hashing algorithm, and 2) application of a sparsity-induced classifier to the compact fingerprints. We test the proposed method on its ability to make a large-scale prediction of compound-protein interactions from compound substructure fingerprints and protein domain fingerprints, and show superior performance of the proposed method compared with the previous chemogenomic methods in terms of prediction accuracy, computational efficiency, and interpretability of the predictive model. All the previously developed methods are not computationally feasible for the full dataset consisting of about 200 millions of compound-protein pairs. The proposed method is expected to be useful for virtual screening of a huge number of compounds against many protein targets.

## Background

The identification of compound-protein interactions is an important part in the drug development toward discovery of new drug leads and new therapeutic protein targets. The completion of the human genome sequencing project has made it possible for us to analyze the genomic space of possible proteins coded in the human genome. At the same time, many efforts have also been devoted to the constitution of molecular databanks to explore the entire chemical space of possible compounds including synthesized molecules or natural molecules extracted from animals, plants, or microorganisms. However, there is little knowledge about the interactions between compounds and proteins. For example, the US PubChem database stores more than 30 million chemical compounds, but the number of compounds with information on their target proteins is very limited [1]. In that field, the importance of chemogenomics research has recently grown fast to investigate the relationship between the chemical space and the genomic space [2,3]. A key issue in chemogenomics is computational prediction of compound-protein interactions on a genome-wide scale.

Recently, a variety of *in silico *chemogenomic approaches have been developed to predict compound-protein interactions or drug-target interactions, assuming that similar compounds are likely to interact with similar proteins. The state-of-the-art in the chemogenomic approach is to built the chemogenomic space of compound-protein pairs as the tensor product of the chemical space of compounds and the genomic space of proteins, and analyze compound-protein pairs by machine learning classifiers such as support vector machine (SVM) [4-8]. However, the input of the SVM method in most previous works is the pairwise kernel similarity matrix of compound-protein *pairs*, which makes it difficult to analyze large-scale data. For example, it is impossible to apply standard implementations such as LIBSVM [9] and SVM*^light^*[10], because it requires prohibitive computational time and the size of the kernel matrix for compound-protein pairs is too huge to construct explicitly in the memory. All previous chemogenomic methods are not suitable for scalable screening of millions of or billions of compound-protein pairs.

Fingerprint is a powerful way to efficiently summarize information about various bio-molecules (e.g., compounds, proteins), that is, encoding their molecular structures or physicochemical properties into finite-dimensional binary vectors. The fingerprint representation has a long history in chemoinformatics, and many 1D, 2D or 3D descriptors for molecules have been proposed [11] and adopted in many molecular databases such as PubChem [1] and ChemDB [12]. The fingerprints can be used for exploring the chemical space based on their Euclidian distance or Tanimoto coefficients, and can also be used as inputs of various machine learning classifiers to predict various biological activities of compounds [13]. The fingerprint representation is applicable to proteins as well [14,15].

In this study we consider representing compound-protein pairs by the fingerprints to use them as inputs of linear SVM, because the linear SVM provides us with interpretable predictive models and works well for super-high dimensional data [16]. A straightforward way is to represent each compound-protein pair by taking the tensor product of the compound fingerprint and the protein fingerprint, which enables biological interpretation of chemogenomic features (functional associations between compound substructures and protein domains) behind interacting compound-protein pairs [8]. However, the resulting fingerprint is sparse and super-high dimensional. Even worse, the total number of fingerprints is the product of the number of compounds and the number of proteins, so it is difficult to train classical linear SVM for extremely large-scale data. Although optimization techniques of linear SVM have recently advanced [17-20], they are not enough to analyze a huge number of compound-protein pairs in practice.

In this paper we develop a novel chemogenomic method to make a scalable prediction of compound-protein interactions from heterogeneous biological data using minwise hashing, which is applicable for virtual screening of a huge number of compounds against many human proteins. The proposed method mainly consists of two steps: 1) construction of new compact fingerprints for compound-protein pairs by an improved minwise hashing algorithm, and 2) application of the linear SVM to the compact fingerprints. A unique feature of the proposed method is that the linear SVM with the compact fingerprints generated by the minwise hashing is able to simulate the nonlinear property of the kernel SVM. We test the proposed method on its ability to make a large-scale prediction of compound-protein interactions from compound substructure fingerprints and protein domain fingerprints, and show superior performance of the proposed method compared with the previous chemogenomic methods in terms of prediction accuracy, computational efficiency, and interpretability of the predictive model. All the previously developed methods are not computationally feasible for the full dataset consisting of about 200 millions of compound-protein pairs.

## Materials

Compound-protein interactions involving human proteins were obtained from the STITCH database [21]. Compounds are small molecules and proteins belong to many different classes such as enzymes, transporters, ion channels, and receptors. The dataset consists of 300,202 known compound-protein interactions out of 216,121,626 possible compound-protein pairs, involving 35,366 compounds and 6,111 proteins. Note that duplicated compounds were removed. The set of known interactions is used as gold standard data.

Chemical structures of compounds were encoded by a chemical fingerprint with 881 chemical substructures defined in the PubChem database [1]. Each compound was represented by a substructure fingerprint (binary vector) whose elements encode for the presence or absence of each of the 881 PubChem substructures by 1 or 0, respectively.

Genomic information about proteins was obtained from the UniProt database [22], and the associated protein domains were obtained from the PFAM database [23]. Proteins in our dataset were associated with 4,137 PFAM domains. Each protein was represented by a domain fingerprint (binary vector) whose elements encode for the presence or absence of each of the retained 4,137 PFAM domains by 1 or 0, respectively.

## Methods

We deal with the in-silico chemogenomics problem as the following machine learning problem: given a set of *n *compound-protein pairs (*C*_1_, *P*_1_),..., (*C_n_, P_n_*), then estimate a function *f*(*C, P*) that would predict whether a compound *C *binds to a protein *P *. In addition, we attempt to estimate an interpretable function *f *in order to extract informative features. Since our dataset consists of about 216 millions of compound-protein pairs, we propose an efficient and general approach to solve these problems.

### Model

Linear models are a feasible tool for large-scale classification and regression tasks such as linear support vector machines (linear SVM) and logistic regression which provide comprehensible models for these tasks. Generally, linear models represent each example *E *as a feature vector Φ(*E*) ∈ ℜ*^D ^*and then estimate a linear function *f*(*E*) = ***w***^T^Φ(*E*) whose sign is used to predict whether or not the example *E *is positive or negative. Note that fingerprints are used for feature vectors in this study. The weight vector ***w ***∈ ℜ*^D ^*is estimated based on its ability to correctly predict the classes of examples in the training set. Since each element of the weight vector ***w ***corresponds to an element of the fingerprint Φ(*E*), we can interpret salient features by sorting elements of Φ(*E*) according to the values of the corresponding elements of ***w***.

In this study each compound-protein pair corresponds to an example. Thus, it is necessary to represent each compound-protein pair (*C, P*) as a single fingerprint Φ(*C, P*) and then estimate a function *f*(*C, P*) = ***w***^T^Φ(*C, P*) whose sign is used to predict whether a compound *C *interacts with a protein *P *or not. As in the previous case, we can extract effective features in Φ(*C, P*) for compound-protein interaction predictions.

### Fingerprint representation of compound-protein pairs

A fingerprint representation of compound-protein pairs has a large impact on not only classification ability of linear models but also interpretability of features. To meet both demands, we represent each compound-protein pair by a fingerprint using the compound fingerprint and the protein fingerprint.

The fingerprint of a compound *C *is represented by a *D*-dimensional binary vector: Φ(*C*) = (*c*_1_, *c*_2_, ..., *c_D_*)^T ^where *c_i _*∈ {0, 1}, *i *= 1, ..., *D*. The fingerprint of a protein *P *is represented by a *D*′-dimensional binary vector as well: Φ(*P*) = (*p*_1_, *p*_2_, ..., *p_D′_*)T where *p_i _*∈ {0, 1}, *i *= 1, ..., *D′*. We define the fingerprint of each compound-protein pair as the tensor product of Φ(*C*) and Φ(*P*) as follows:

$$\begin{array}{cc} \Phi \left(C,P\right) & =\Phi \left(C\right)⊗\Phi \left(P\right)\\ & ={\left(c_{1}p_{1},\ldots ,c_{1}p_{D^{\prime }},\ldots ,c_{D}p_{1},\ldots ,c_{D}p_{D^{\prime }}\right)}^{T}. \end{array}$$

Φ(*C, P*) consists of all possible products of elements in two fingerprints Φ(*C*) and Φ(*P*), so the fingerprint is a *D *× *D′ *dimensional binary vector. The dimensions of Φ(*C*), Φ(*P*), and Φ(*C, P*) in this study are *D *= 881, *D′ *= 4, 137, and *DD′ *= 3, 644, 697, respectively.

### Minwise hashing

We propose to use *minwise hashing *for analyzing fingerprints efficiently. In this section, we make a brief review of minwise hashing [24]. A key observation is that any fingerprint can be represented by a set uniquely. Each fingerprint Φ(*C, P*) is represented by a set *S *⊆ Ω = {1, 2, ..., *D *× *D′*}. Given two sets *S_i _*and *S_j_*, Jaccard similarity *J*(*S_i_, S_j_*) of *Si *and *S_j _*is defined as

$$J\left(S_{i},S_{j}\right)\;=\;\frac{\left|S_{i}\cap S_{j}\right|}{\left|S_{i}\cup S_{j}\right|}\;\;\;\;\;\left(i,\;j\;=\text{ 1},\text{ 2},\cdot \cdot \cdot ,\;n\right).$$

Minwise hashing is a random projection of sets such that the expected Hamming distance of obtained symbol strings is proportional to the Jaccard similarity [24]. We pick *ℓ *random permutations $\pi_{k}$, *k *= 1, ..., *ℓ*, each of which maps [1, *M*] to [1, *M*]. Let *T_i _*= *t_i1_*, ⋯, *t_iℓ _*be a resultant string projected from *S_i_*. The projection is defined as the minimum element of the random permutation of the given set,

$$t_{ik}\;=\;\text{ min}\left\{\pi_{\text{k}}\left(S_{i}\right)\right\}$$

For example, if $\pi_{k}$ is defined as

$$\left(\text{1},\text{ 2},\text{ 3},\text{ 4},\text{ 5},\text{ 6},\text{ 7},\text{ 8}\right)\to \left(\text{3},\text{ 8},\text{ 7},\text{ 1},\text{ 2},\text{ 6},\text{ 4},\text{ 5}\right),$$

*S_i _*= (1, 4, 6, 7) is transformed to $\pi_{k}$(*S_i_*) = (3, 1, 6, 4), and the final product is *t_ik _*= 1. The collision probability, which is a probability that two sets *S_i _*and *S_j _*are projected to the same elements *t_ik _*and *t_jk_*(*t_ik _= t_jk_*), is described as

$$\text{Pr}\left[t_{ik}=t_{jk}\right]=J\left(S_{i},S_{j}\right).$$

Therefore, the expected Hamming distance between *t_i _*and *t_j _*is identical to *ℓ*(1 - *J*(*S_i_, S_j_*)).

### Saving memory by additional hashing

The common practice of minwise hashing is to store each hashed value using 64bits [24]. The storage (and computational) cost is prohibitive in large-scale applications. To overcome this problem, Li et al. proposed *b*-bit minwise hashing [25,26], which rounds each hashing value to only lower *b*-bits value. However, a theoretical analysis of the collision probability is complicated.

Here we introduce a simple yet effective method such that a theoretical estimation of collision probability can easily be derived. In our method, the hashing values are further hashed to a set {1, ..., *N*} randomly, where *N *<<*M*. This projection is defined as follows:

$$s_{ik}=h\left(t_{ik}\right),$$

where *h *: {1, ..., *M*} → {1, ..., *N*} is a random hash function. If *t_ik _*and *t_jk _*are identical, *s_ik _*and *s_jk _*always collide. If not, they collide with probability 1/*N*. Thus, the collision probability is obtained as follows:

(1) $$Pr\left[s_{ik}\;=\;s_{jk}\right]\;=\;1-\frac{N-1}{N}\left(1-J\left(S_{i},\;S_{j}\right)\right).$$

**Figure 1:**
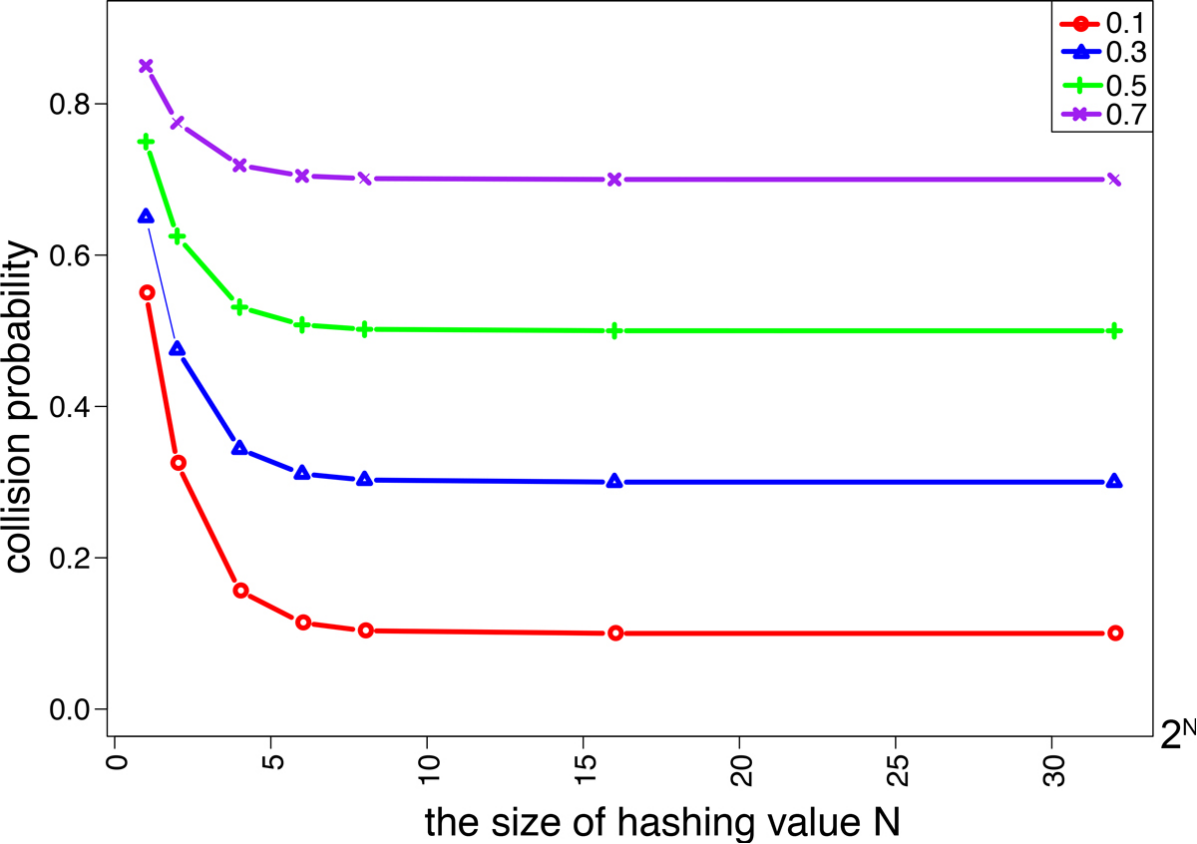
Collision probabilities for varying the size of additional hashing value *N*.

Figure 1 shows collision probability for each hashing value, where four different Jaccard similarities, 0.1, 0.3, 0.5 and 0.7, are chosen. It is observed that collision probabilities do not increase for hashing values of no less than 2^8^. Thus, small hashing values can be chosen without loss of accuracy.

### Building compact fingerprints by minwise hashing

Learning linear models with large-scale high-dimensional data is a difficult problem in terms of computational cost. Here we propose a method to represent the original fingerprint of compound-protein pair by a new fingerprint whose size is smaller than that of the original fingerprint.

A crucial observation is that any fingerprint can be represented as a set uniquely, and can also be converted into a string uniquely. First, we convert the original fingerprint of each compound-protein pair into a string by applying minwise hashing and additional hashing. Next, we expand hashing values organizing the string into a new binary vector whose dimension is much smaller than that of the original fingerprint.

**Figure 2:**
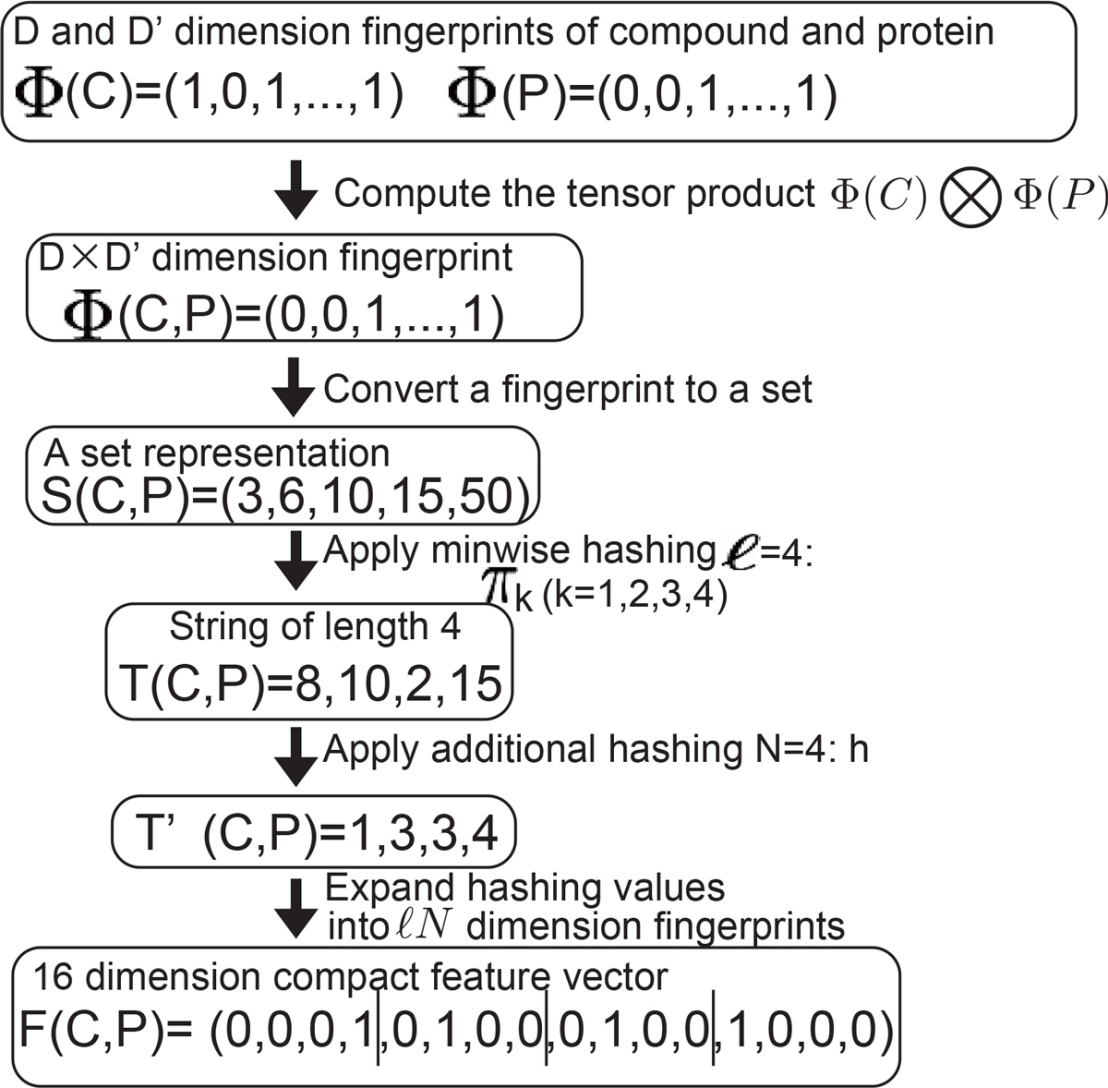
**Construction of a compact fingerprint**. The vertical bars in *F*(*C, P*) are inserted for readability. Each range represented by the vertical bars in *F*(*C, P*) includes elements expanded from a hashing value.

Let ***S***(*C, P*) be a set representation of Φ(*C, P*) where *i *is contained in ***S***(*C, P*) iff the *i*-th element of Φ(*C, P*) is 1. We apply minwise hashing *π_k_*(*k *= 1, ..., *ℓ*) to ***S***(*C, P*) to generate a string ***T***(*C, P*) = *t*_1_, *t*_2_, ..., *t_ℓ_*, where each element *t_k _*takes a value ranging from 1 to *M*. We additionally hash each element *t_k _*to a new small value $t_{k}^{\prime }$ ranging from 1 to *N*(*N *<<*M*) by applying additional hash h, and generate a new string $T^{\prime }\left(C,P\right)\;=\;t_{1}^{\prime },\;t_{2}^{\prime },\;\ldots ,t_{\ell }^{\prime }$. Each value $t_{k}^{\prime }$ in the string ***T′***(*C, P*) is expanded to an *N*-dimensional binary vector *f_k_*, where the $t_{k}^{\prime }$-th element is 1 and the others are 0. Finally, we concatenate *f*_1_, ..., *f_ℓ _*into a single one, and obtain an *ℓN*-dimension binary vector ***F***(*C, P*) = (*f*_1_, ..., *f_ℓ_*). The newly obtained ***F***(*C, P*) is referred to as "compact fingerprint". Figure 2 shows an illustration of the proposed procedure.

### Linear support vector machines (Linear SVM)

We use linear SVM as a classifier. The predictive model is typically learned by minimizing objective functions with a regularization. The most common regularization is *L*_2_-regularization which keeps most elements in the weight vector to be non-zeros, so one suffers from difficulty in interpreting the predictive model with many non-zero weights. *L*_2_-regularized linear SVM is referred to as L2SVM. Another regularization is *L*_1_-regularization which keeps most elements in the weight vector to be zeros, so the *L*_1_-regularization is popularly used for its high interpretability owing to the induced sparsity. *L*_1_-regularized linear SVM is referred to as L1SVM.

Given a training set of compound-protein pairs and labels ${\left\{F\left(C_{i},P_{i}\right),\;y_{i}\right\}}_{i=1}^{n},\;y_{i}\in \left\{+\text{1},\;-\text{1}\right\}$, linear SVM is formulated as the following unconstrained optimization problem:

(2) $$\underset{w}{\text{min}}\overset{n}{\underset{i=1}{\sum }}\text{max}\left\{1-y_{i}w^{\text{T}}F\left(C_{i},\;P_{i}\right),\;0\right\}.$$

To prevent overfitting, the weight vector is optimized with *L*_1_-regularization and *L*_2_-regularization as follows:

(3) $$\underset{w}{\text{min}}{||w||}_{1}+\overset{n}{\underset{i=1}{\sum }}\text{max}\left\{1-y_{i}w^{\text{T}}F\left(C_{i},\;P_{i}\right),\;0\right\}.$$

and

(4) $$\underset{}{\underset{w}{\text{min}}{||w||}_{2}+}\overset{n}{\underset{i=1}{\sum }}\text{max}\left\{1-y_{i}w^{\text{T}}F\left(C_{i},\;P_{i}\right),\;0\right\}.$$

where ||⋯||_1 _and ||⋯||_2 _are *L*_1 _and *L*_2 _norms, and *C *is a hyper-parameter. Recently, optimization algorithms for linear SVM have rapidly advanced. In this study, we use an efficient optimization algorithm named LIBLINEAR [18]^1^.

^1^The software is available from http://www.csie.ntu.edu.tw/~cjlin/liblinear/

In our method, we propose to use the compact fingerprint ***F***(*C, P*) instead of the original fingerprint Φ(*C, P*) as an input for L1SVM and L2SVM. L1SVM and L2SVM with the compact fingerprints ***F***(*C, P*) are referred to as Minwise Hashing-based L1SVM (MH-L1SVM) and Minwise Hashing-based L2SVM (MH-L2SVM), respectively. In contrast, L1SVM and L2SVM with the original fingerprints Φ(*C, P*) are referred to as L1SVM and L2SVM, respectively, which correspond to previous methods [8].

In most previous works the kernel SVM method was used, but the input of kernel SVM is the kernel similarity matrix for compound-protein pairs [5,6], which makes it difficult to apply the kernel SVM to large-scale interaction prediction. This is because the time complexity of the quadratic programming problem for kernel SVM is $O\left(n_{c}^{3}\;\times \;n_{p}^{3}\right)$, where *n_c _*is the number of compounds and *n_p _*is the number of proteins, and the space complexity is $O\left(n_{c}^{2}\;\times \;n_{p}^{2}\right)$, which is just for storing the kernel matrix. Moreover, kernel SVM does not have any interpretability of the predictive model because it is not able to extract features.

### Relation to kernel SVM

In this section, we describe a theoretical foundation for using linear SVM with compact fingerprints and discuss the relation to kernel SVM [5,6]. Kernel matrix is an *n *× *n *matrix ***K ***satisfying $\sum_{ij}c_{i}c_{j}K_{ij}\;\leq \;0$ for all real vectors *c*. Such a property is called positive definite (PD), which is necessary to effectively train an SVM classifier with a kernel matrix. A matrix **A **is PD if it can be written as an inner product of matrices **B**^T^**B**.

Our linear SVM with compact fingerprints simulates non-linear SVMs with the Jaccard similarity matrix for the following reasons.

1. Each element of the pairwise kernel matrix of compound-protein pairs is defined as the number of common elements between two sets ***S***(*C, P*) and ***S***(*C′, P′*), i.e, |***S***(*C, P*) ∩ ***S***(*C′, P′*)|. The pairwise kernel matrix is PD. Jaccard similarity is a pairwise kernel normalized by the cardinality of the union of two sets ***S***(*C, P*) and ***S***(*C′, P′*), i.e., |***S***(*C, P*) ∪ ***S***(*C′, P′*)|. The Jaccard similarity matrix of compound-protein pairs, where each element is Jaccard similarity of two sets ***S***(*C, P*) and ***S***(*C′, P′*), is also PD.

2. Let the minwise hashing matrix of compound-protein pairs be a matrix whose element is defined as the inner product of two compact fingerprints ***F***(*C, P*) and ***F***(*C′, P′*). The minwise hashing matrix is PD.

3. The (*i, j*)-element of the Jaccard similarity matrix correlates with the (*i, j*)-element of the minwise hashing matrix.

4. While Jaccard similarity is a non-linear function, the inner product is a linear function.

The third reason is true because the collision probability, which is a probability that two minwise hashing and additional hashing values for two sets ***S***(*C, P*) and ***S***(*C′, P′*) are the same, is positively correlated with Jaccard similarity ***J***(***S***(*C, P*), ***S***(*C′, P′*)) (Equation 1).

### Feature extraction for biological interpretation

Extracting informative features in the original fingerprint for predicting compound-protein interactions is also an important task. Since each value of the weight vector in a linear model corresponds to the importance of the corresponding feature of the original fingerprint in the classification task. In our method, we apply minwise hashing and additional hashing to the original fingerprint, and build the compact fingerprint to efficiently train a linear SVM classifier. Thus, it is not trivial to extract features in the original fingerprint in our framework.

We propose to keep inverse mappings $\pi_{k}^{-1}$ and *h*^-1 ^for permutation $\pi_{k}$ and additional hashing *h*, and apply *h*^-1 ^and $\pi_{k}^{-1}$ to each element in the compact fingerprint in order to recover the weight vector for the original fingerprint. Let $\pi_{k}^{-1}$ : [1, *M*] → [1, *M*] (*k *= 1, ..., *ℓ*) be an inverse mapping for permutation *π_k _*: [1, *M*] → [1, *M*]. Let *h*^-1 ^: [1, *N*] → [1, *M*]^∗ ^be an inverse mapping for additional hashing *h *: [1, *M*] → [1, *N*]. Note that *h*^-1 ^is, basically, a one-to-many mapping *N *<<*M*.

**Figure 3:**
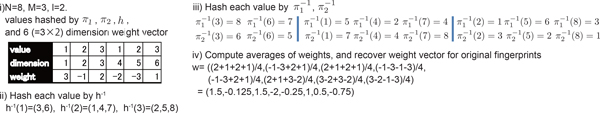
**Recovery of a weight vector**.

First, we apply inverse mapping *h*^-1 ^to each element in the compact fingerprint to recover values hashed by additional hashing h. Since *h*^-1 ^is a one-to-many mapping, several values are recovered. Then, inverse mapping π^-1 ^is applied to each value in order to recover an element in the original fingerprint. Finally, we compute an average of the weights learned by linear SVMs, which provides the recovered weight vector for the original fingerprint. Figure 3 shows an illustration of the proposed procedure.

## Results

### Performance evaluation

We tested MH-L1SVM and MH-L2SVM (newly proposed methods) on their abilities to predict compound-protein interactions from compound substructure fingerprints and protein domain fingerprints, and compared the performance with L1SVM and L2SVM (previous methods [8]) in terms of prediction accuracy and computational cost. Note that the kernel SVM (the state-of-the-art [4-7]) was not computationally feasible for our large data. Our full dataset is too huge (consists of about 216 millions of compound-protein pairs), so we used a subset of the full data for efficient evaluation of the four different methods. In the sub-dataset, the numbers of positive and negative examples were balanced, i.e., 300,202, respectively and 600,404 in total. We performed two types of 5-fold cross-validations: pair-wise cross-validation and block-wise cross-validation.

In the pair-wise cross-validation we perform the following procedure: 1) We randomly split compound-protein pairs in the gold standard set into five subsets of roughly equal sizes, and take each subset in turn as a test set. 2) We train a predictive model on the remaining four subsets. 3) we compute the prediction scores for compound-protein pairs in the test set. 4) Finally, we evaluate the prediction accuracy over the five folds. The pair-wise cross-validation assumes the situation where we want to detect missing interactions between known ligand compounds and known target proteins with information about interaction partners. In the block-wise cross-validation we perform the following procedure: 1) We randomly split compounds and proteins in the gold standard set into five compound subsets and five protein subsets, and take each compound subset and each protein subset in turn as test sets. 2) We train a predictive model on compound-target pairs in the remaining compound subsets and four protein subsets. 3) We compute the prediction scores for compound-protein pairs involving test compound set and test protein set. 4) Finally, we evaluate the prediction accuracy over the five folds. The block-wise cross-validation assumes the situation where we want to detect new interactions for newly arriving ligand candidate compounds and target candidate proteins with no information about interaction partners. In the both cases, we evaluated the performance by the area under the ROC curve (AUC) and execution time. The cross-validations were performed by varying the hyper-parameter *C *= 10^-5^, 10^-4^, ..., 10^5 ^and chosen as the one to achieve the best AUC score.

**Figure 4:**
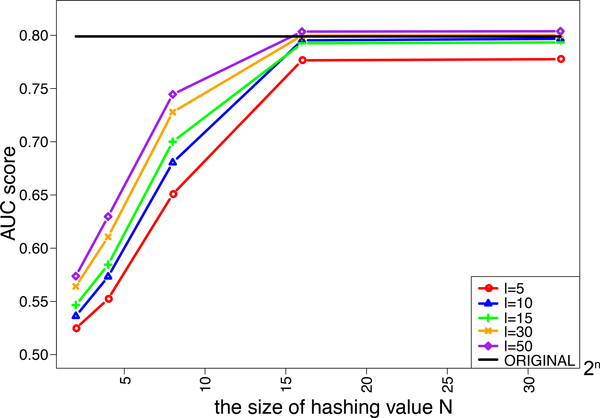
**AUC score of L1SVM for varying the size of additional hashing value *N***.

**Figure 5:**
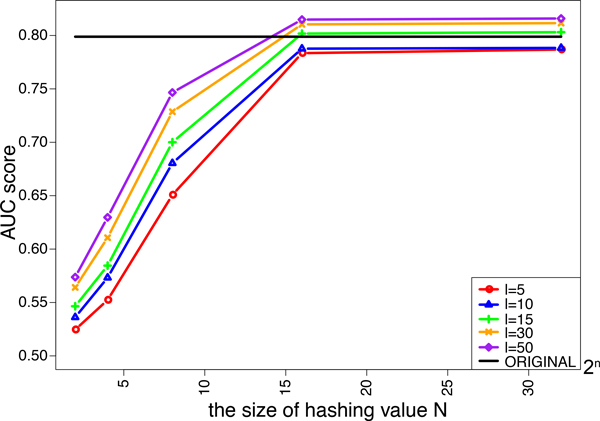
AUC score of L2SVM for varying the size of additional hashing value *N*.

We investigated the effects of the length of strings *l *and the size of hashing values *N *in the minwise hashing process of MH-L1SVM and MH-L2SVM on the performance. We tried five different lengths of string *ℓ *= 5, 10, 15, 30, 50. The size of additional hashing values *N *is varied from 2^2 ^to 2^32^. Figure 4 and 5 shows the AUC scores for MH-L1SVM and MH-L2SVM in the pair-wise cross validation. It was observed that the AUC scores reached the maximum with the length of string *ℓ *= 10 and the size of additional hashing value *N *= 2^16^, and the AUC score was comparable to that for the original fingerprint.

**Figure 6:**
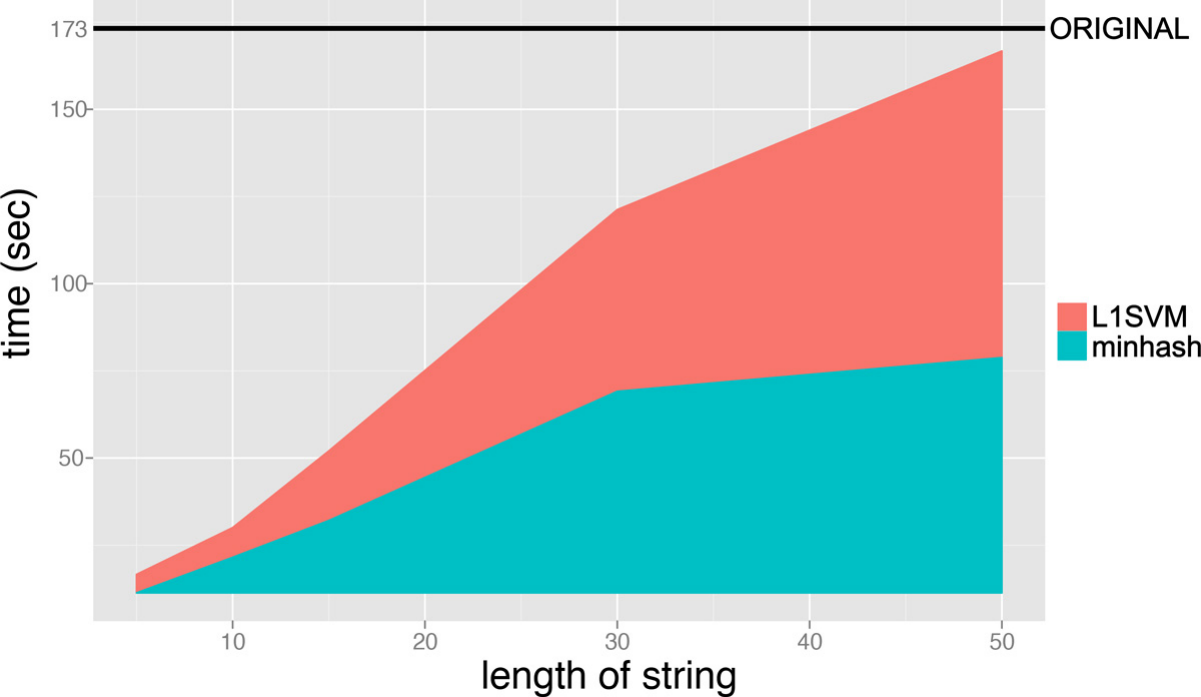
Learning time of L1SVM for fixing the size of additional hashing value *N *= 2^16 ^and varying the length of string *ℓ*.

**Figure 7:**
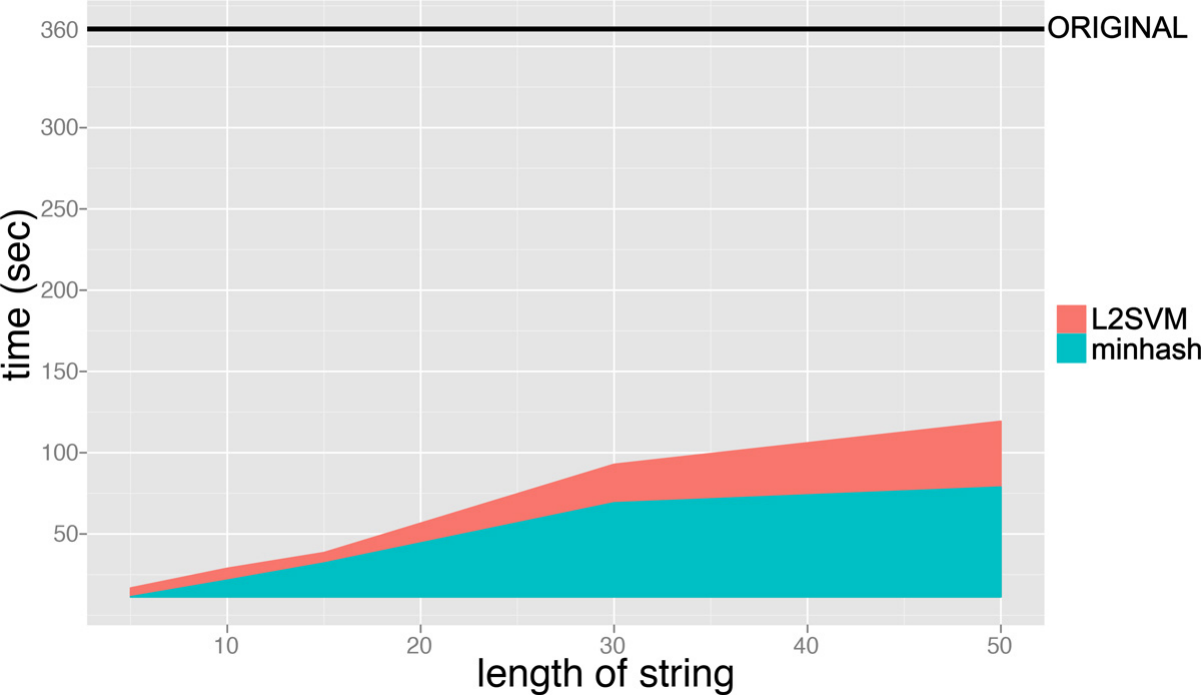
Learning time of L2SVM for fixing the size of additional hashing value *N *= 2^16 ^and varying the length of string *ℓ*.

Figure 6 and 7 shows the execution time for performing the minwise hashing and for learning SVM classifiers, where the length of string *ℓ *is varied from 5 to 50 and the size of additional hashing value is fixed to *N *= 2^16^. The AUC scores of MH-L1SVM and MH-L2SVM with the length of string *ℓ *= 10 and the size of additional hashing *N *= 2^16 ^were comparable to those of L1SVM and L2SVM. In addition, MH-L1SVM and MH-L2SVM achieved certain speedup compared with L1SVM and L2SVM.

**Figure 8:**
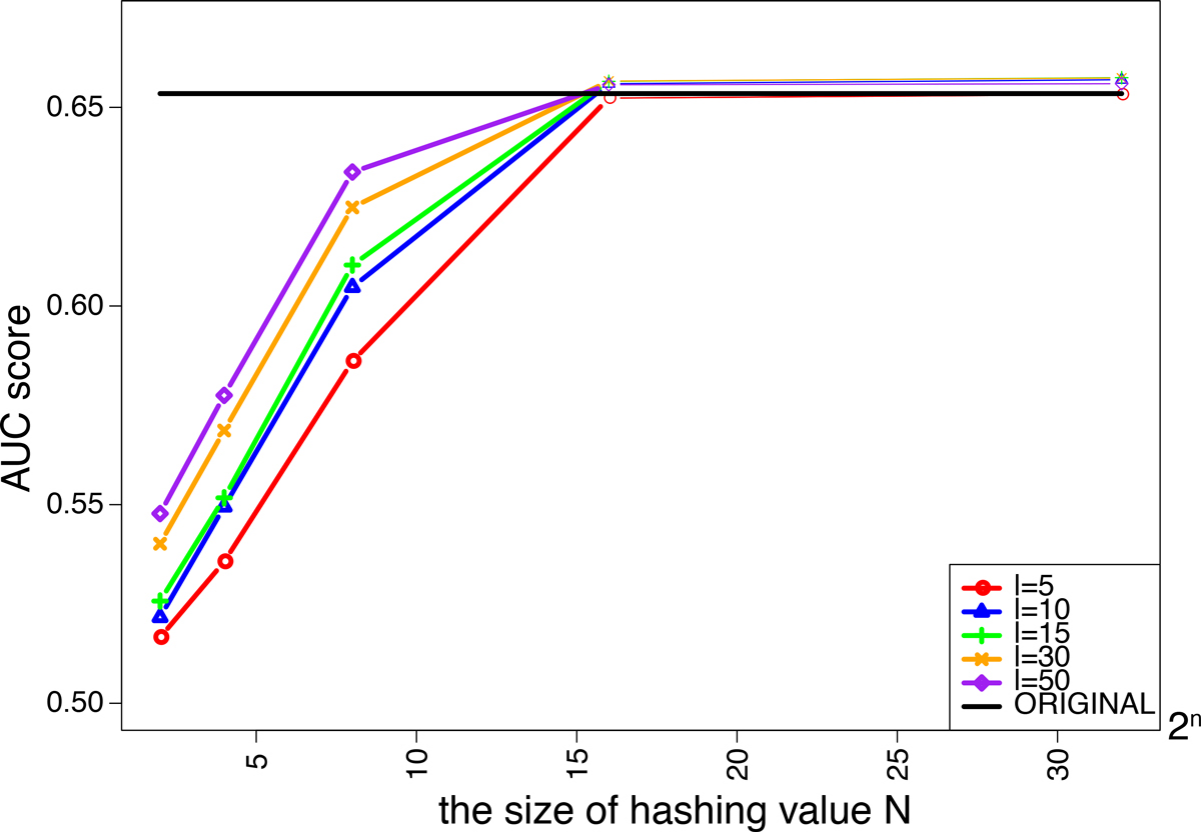
AUC score of L1SVM for varying the size of additional hashing value *N*.

**Figure 9:**
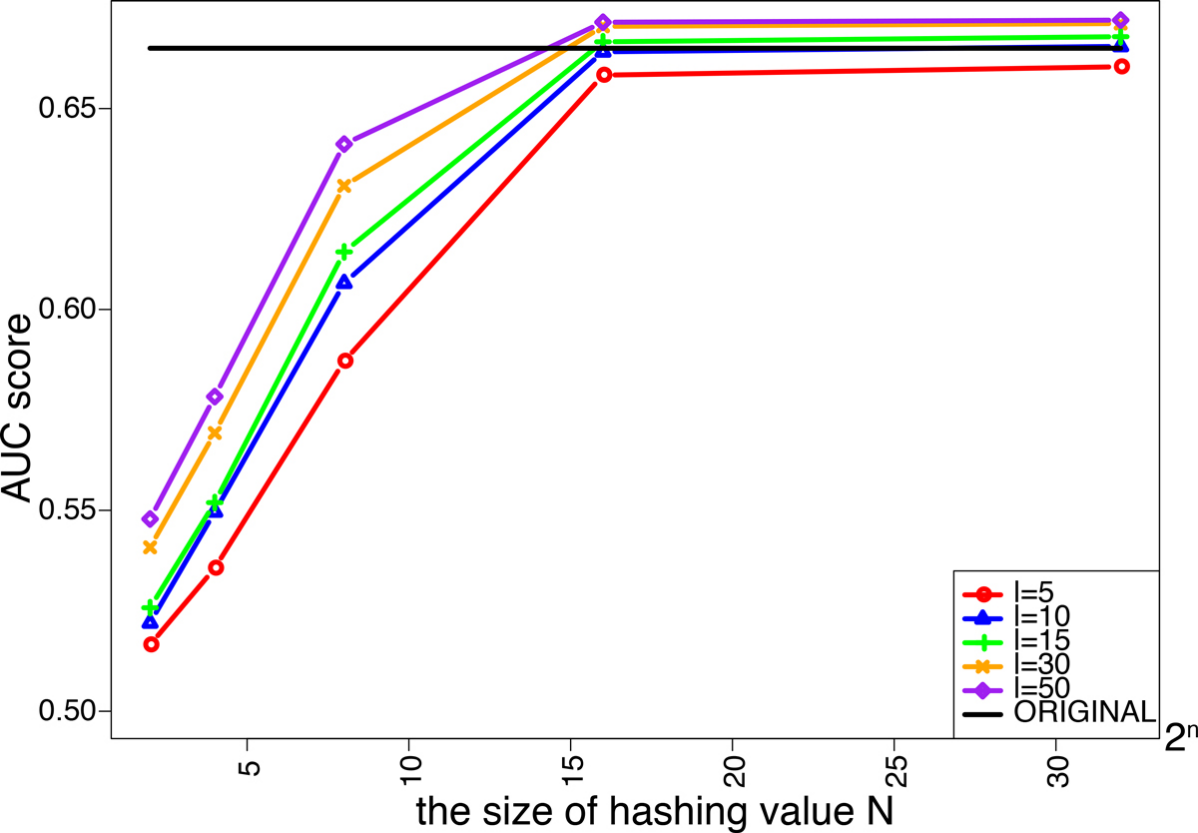
AUC score of L2SVM for varying the size of additional hashing value *N*.

**Figure 10:**
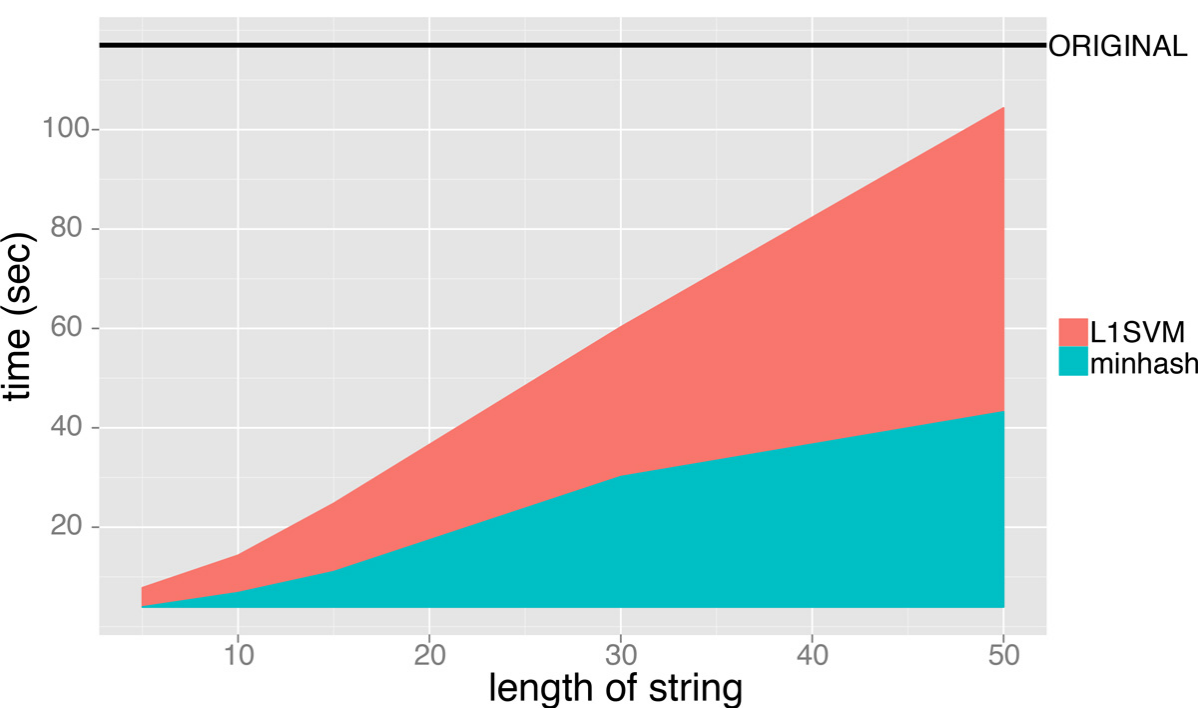
**Learning time of L1SVM for fixing the size of additional hashing value 2^16 ^and varying the length of string**.

**Figure 11:**
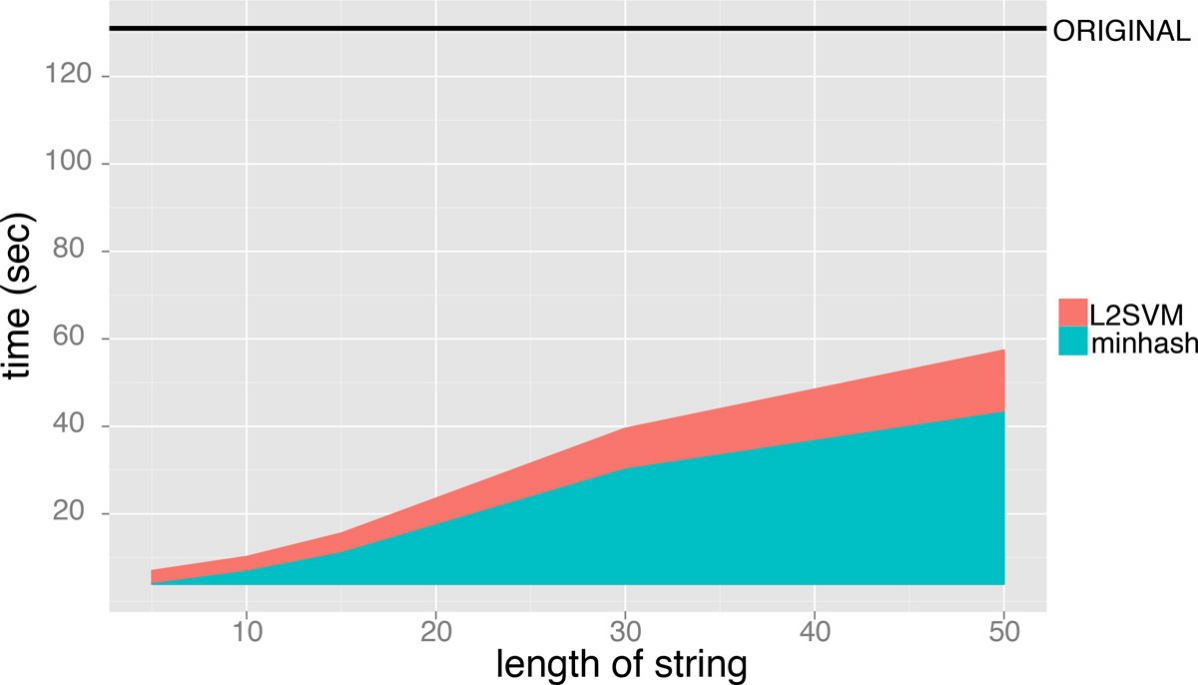
**Learning time of L2SVM for fixing the size of additional hashing value 2^16 ^and varying the length of string**.

The same trends of these results in the pair-wise cross-validation were observed in the case of the block-wise cross-validation as well. The corresponding results for the block-wise cross-validation are shown in Figures 8, 9, 10 and 11. The AUC scores in the block-wise cross-validation were lower than those in the pair-wise cross-validation, which implies that predicting unknown interactions for newly coming compounds and proteins outside of the learning set is much more difficult than detecting missing interactions between compounds and proteins in the learning set.

### Experiments on large-scale datasets

We evaluated the performance for the full data consisting of 216,121,626 compound-protein pairs, where the best parameter values for each method in the cross-validation experiments in the previous subsection were used. We examined the effect of the ratio of positive compound-protein pairs against negative compound-protein pairs on the performance. Note that the number of negative examples is much larger than that of positive examples in our dataset. We varied the number of negative examples in the cross-validation from the same number of positive examples to the number of all possible negative examples.

**Figure 12:**
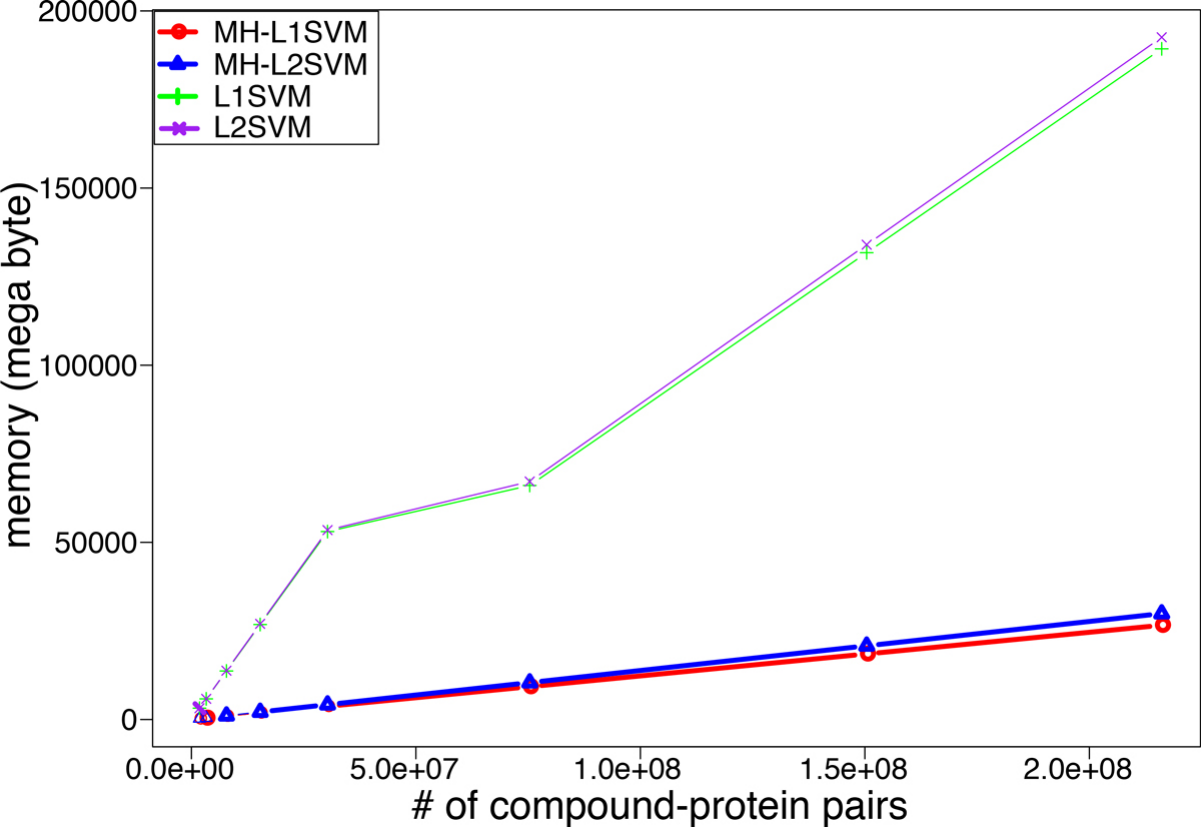
**Memory usage for increasing the number of compound-protein pairs**.

Figure 12 shows the memory usages of the four different methods. It was observed that the memory usage grew linearly as the number of compound-protein pairs increased in each method. Especially, both L1SVM and L2SVM required about 200GB in memory. On the other hand, MH-L1SVM and MH-L2SVM took only about 30GB in memory. There is little difference of memory usage between L1-regularization and L2-regularization.

Table 1 shows the AUC scores in the pair-wise cross-validation. It was observed that the AUC scores of MH-L1SVM and MH-L2SVM were comparable to those of L1SVM and L2SVM, respectively. Table 2 shows training time on the pair-wise cross-validation, where the training time includes the minwise hashing process and the upper limitation is put on the execution time for all methods to 24 hours. MH-L1SVM and MH-L2SVM are significantly faster than L1SVM and L2SVM, respectively. Especially, MH-L2SVM is about 10 times faster than L2SVM. L1SVM did not finish the computation for such a large number of compound-protein pairs within 24 hours. On the other hand, our MH-L1SVM finished the computation and took only 25,060 seconds on average.

**Table 1:** AUC score on pair-wise cross validation experiments

Ratio	Number	MH-L1SVM	MH-L2SVM	L1SVM	L2SVM
1	600, 404	0.78 ± 2.31 × 10^-6^	0.79 ± 2.31 × 10^-6^	0.79 ± 3.22 × 10^-6^	0.80 ± 4.97 × 10^-6^
5	1, 801, 212	0.79 ± 7.23 × 10^-7^	0.80 ± 8.30 × 10^-7^	0.81 ± 2.04 × 10^-7^	0.81 ± 2.04 × 10^-7^
10	3, 302, 222	0.79 ± 1.84 × 10^-6^	0.80 ± 1.35 × 10^-6^	0.81 ± 5.34 × 10^-7^	0.81 ± 4.31 × 10^-7^
25	7, 805, 252	0.79 ± 2.89 × 10^-7^	0.80 ± 6.28 × 10^-8^	0.81 ± 9.87 × 10^-8^	0.81 ± 1.30 × 10^-7^
50	15, 310, 302	0.79 ± 3.21 × 10^-7^	0.81 ± 3.79 × 10^-7^	0.81 ± 3.40 × 10^-8^	0.81 ± 1.72 × 10^-7^
100	30, 320, 402	0.79 ± 2.38 × 10^-7^	0.81 ± 1.49 × 10^-7^	-	0.81 ± 2.43 × 10^-7^
250	75, 350, 702	0.79 ± 2.91 × 10^-7^	0.81 ± 2.42 × 10^-7^	-	0.81 ± 3.66 × 10^-7^

The number of negative examples is varied from the same number of positive examples to the number of negative examples 250 times larger than the number of all positive examples.

**Table 2:** Training time on pair-wise cross validation experiments

Ratio	Number	MH-L1SVM	MH-L2SVM	L1SVM	L2SVM
1	600, 404	29 ± 1	28 ± 1	188 ± 32	387 ± 63
5	1, 801, 212	172 ± 5	38 ± 2	1, 655 ± 156	963 ± 81
10	3, 302, 222	448 ± 41	261 ± 7	1, 261 ± 579	10, 798 ± 1, 981
25	7, 805, 252	1, 808 ± 181	732 ± 17	20,067 ± 1,453	4, 623 ± 782
50	15, 310, 302	1,140 ± 90	811 ± 41	58, 045 ± 5, 678	8, 936 ± 1, 412
100	30, 320,402	7, 601 ± 627	1,643 ± 50	> 24hours	16, 608 ± 2, 732
250	75, 350, 702	25,060 ± 12,417	4,631 ± 795	> 24hours	43, 843 ± 7, 200

The training time includes minwise hashing time. The number of negative examples is varied from the same number of positive examples to the number of negative examples 250 times larger than the number of all positive examples.

The same trends for these results in the pair-wise cross-validation were observed in the block-wise cross-validation as well (See Tables 3 and 4).

**Table 3:** AUC scores on block-wise cross validation experiments

Ratio	Number	MH-L1SVM	MH-L2SVM	L1SVM	L2SVM
1	600, 404	0.66 ± 0.00	0.66 ± 0.01	0.65 ± 0.01	0.67 ± 0.01
5	1, 801, 212	0.66 ± 0.01	0.66 ± 0.01	0.66 ± 0.01	0.67 ± 0.01
10	3, 302, 222	0.66 ± 0.01	0.67 ± 0.01	0.66 ± 0.01	0.67 ± 0.01
25	7, 805, 252	0.66 ± 0.01	0.66 ± 0.01	0.65 ± 0.01	0.66 ± 0.01
50	15, 310, 302	0.66 ± 0.01	0.66 ± 0.01	0.65 ± 0.01	0.66 ± 0.01

The number of negative examples is varied from the same number of positive examples to the number of negative examples 50 times larger than the number of all positive examples.

**Table 4:** Training times on block-wise cross validation experiments

Ratio	Number	MH-L1SVM	MH-L2SVM	L1SVM	L2SVM
1	600, 404	8 ± 0	7 ± 1	131 ± 7	117 ± 28
5	1, 801, 212	76 ± 9	31 ± 1	982 ± 184	252 ± 30
10	3, 302, 222	237 ± 23	55 ± 4	3925 ± 92	475 ± 93
25	7, 805, 252	582 ± 79	107 ± 4	2606 ± 265	322 ± 20
50	15, 310, 302	1889 ± 82	243 ± 8	7133 ± 664	729 ± 18

The training time includes minwise hashing time. The number of negative examples is varied from the same number of positive examples to the number of negative examples 50 times larger than the number of all positive examples.

Table 5 shows the AUC scores and training times in using all possible negative examples, where only 1-fold of the 5-fold cross-validation was performed on this dataset. On this extremely large data, L1SVM and L2SVM did not finish the computation within 24 hours. On the other hand, MH-L1SVM and MH-L2SVM finished the computation, and the AUC scores were reasonable. The training times of MH-L1SVM and MH-L2SVM were 157,013 and 10,054 seconds, respectively. These results suggest the usefulness of our proposed methods in large-scale applications.

**Table 5:** AUC score and training time on the full data consisting of all 216,121,626 compound-protein pairs

Measure	MH-L1SVM	MH-L2SVM	L1SVM	L2SVM
AUC score	0.79	0.81	-	-
Training Time (sec)	157, 013	10, 054	> 24hours	> 24hours

**Figure 13:**
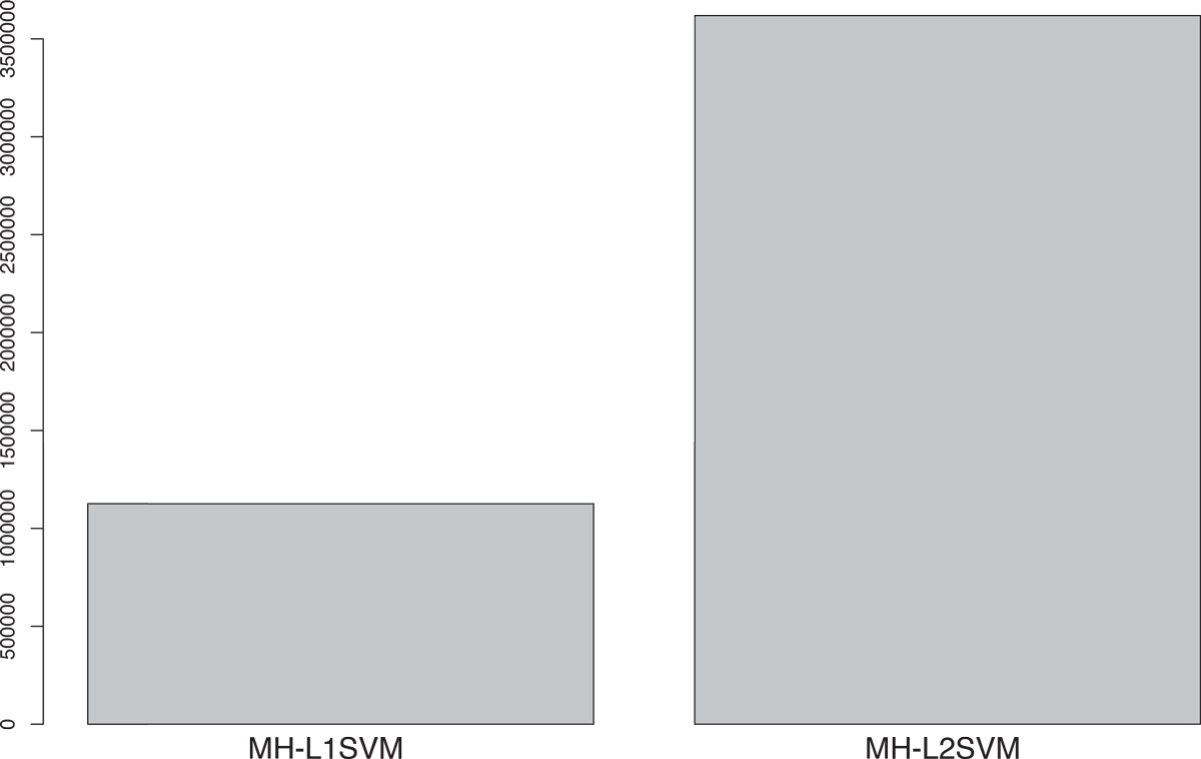
**The number of features extracted by MH-L1SVM and MH-L2SVM**.

Figure 13 shows the numbers of features extracted by MH-L1SVM and MH-L2SVM. The number of features extracted by MH-L1SVM are about third times smaller than that of features extracted by MH-L2SVM. This result suggests that MH-L1SVM provides us with more selective features, which would help to make a biological interpretation about the functional associations between compound substructures and protein domains behind compound-protein interactions.

## Discussion and conclusion

In this paper we proposed a novel chemogenomic method to predict unknown compound-protein interactions on a large scale, which was made possible by using an improved minwise hashing algorithm to efficiently represent the fingerprints of compound-protein pairs. Interestingly, the linear SVM with the compact fingerprints generated by the minwise hashing is able to simulate the nonlinear property of the kernel SVM (the state-of-the-art). The originality of the proposed method lies in the scalable prediction of compound-protein interactions, in the computational efficiency, and in the interpretability of the predictive model. It should be pointed out that all previous methods were not computationally feasible for the full data. The proposed method is expected to be useful for virtual screening of a large number of compounds against many protein targets.

The proposed method can be used, as soon as compounds and proteins are represented by binary descriptors (chemical substructures and protein domains in this study). However, a limitation of the proposed method is that the performance depends on the definitions of chemical substructures of compounds and functional domains of proteins. The use of other descriptors (e.g., KlekotaRoth, ECFP6, Daylight, and Dragon) could improve the generalization properties of the method. Datasets, all results and softwares are available at https://sites.google.com/site/interactminhash/.

## Competing interests

None declared.

## Authors' contributions

YT implemented the algorithm of the methods, made all analyses, and drafted the manuscript. YY prepared the datasets, and drafted the manuscript.

## Declarations

The publication cost for this work was supported by the PRESTO program of the Japan Science and Technology Agency (JST).

This article has been published as part of *BMC Systems Biology *Volume 7 Supplement 6, 2013: Selected articles from the 24th International Conference on Genome Informatics (GIW2013). The full contents of the supplement are available online at http://www.biomedcentral.com/bmcsystbiol/supplements/7/S6.
